# Precision medicine in peripartum cardiomyopathy: advancing diagnosis and management through genomic and phenotypic integration

**DOI:** 10.1097/MS9.0000000000002329

**Published:** 2024-07-01

**Authors:** Ajeet Singh, Hamza Irfan, Tooba Ali, Sanila Mughal, Ayesha Shaukat, Mohammad Jawwad, Aymar Akilimali

**Affiliations:** a Department of Internal Medicine, Dow University of Health Sciences, Karachi; b Department of Medicine, Shaikh Khalifa Bin Zayed Al Nahyan Medical and Dental College, Lahore, Pakistan; c Department of research, Medical Research Circle (MedReC), Bukavu, Democratic Republic of Congo

**Keywords:** biomarker evaluation, genetic testing, peripartum cardiomyopathy, personalized medicine, ventricular systolic dysfunction

## Abstract

Peripartum cardiomyopathy (PPCM) is a rare and life-threatening cardiac condition characterized by heart failure due to left ventricular systolic dysfunction, often developing in late pregnancy or the early postpartum period. Despite being a leading cause of maternal morbidity and mortality, clinical presentation of PPCM frequently overlaps with normal pregnancy-related physiological changes, causing diagnostic delays and increased complications. Current management strategies, primarily derived from general heart failure protocols, are evolving to address the unique aspects of PPCM. This includes the development of personalized medicine approaches that integrate genetic profiling, biomarker evaluation, and clinical phenotyping. Notable genes such as titin (TTN), Bcl2-associated athanogene 3 (BAG3), and lamin A/C (LMNA) are implicated in PPCM, revealing a complex genetic landscape similar to other cardiomyopathies. Biomarkers like N-terminal pro-brain-type natriuretic peptide (NT-proBNP) and cardiac troponin T (cTnT) are under investigation for their diagnostic and prognostic value, indicating that personalized treatments hold the promise of enhancing diagnostic precision and therapeutic outcomes by tailoring interventions to individual patient profiles. This review article aims to highlight how integrating genetic and phenotypic data can establish a novel framework for managing PPCM, potentially transforming treatment paradigms and improving long-term outcomes.

## Introduction

Highlights

- Peripartum cardiomyopathy (PPCM) symptoms mimic normal pregnancy-related changes, leading to diagnostic delays and increased risks of complications such as thromboembolism, maternal and neonatal mortality, and exacerbated left ventricular dysfunction.
- Genetic profiling reveals a complex landscape with key genes like titin (TTN), Bcl2-associated athanogene 3 (BAG3), and lamin A/C (LMNA) implicated in PPCM, suggesting genetic predispositions and shared pathways with other cardiomyopathies.
- While standard heart failure treatments include diuretics, beta-blockers, and mineralocorticoid receptor antagonists, with adjustments for pregnancy and breastfeeding safety, personalized treatment approaches (including genetic profiling and biomarker evaluations) show potential for revolutionizing the management of PPCM and improve patient outcomes.
- PPCM management requires a comprehensive, personalized approach leveraging precision medicine, genomic technologies, and biomarker identification to improve diagnostic precision and treatment outcomes.

Peripartum cardiomyopathy (PPCM) is a rare, idiopathic, and life-threatening complication of pregnancy characterized by the development of heart failure secondary to left ventricular systolic dysfunction, with reduced LV ejection fraction (LVEF <45%), in the absence of any other identifiable cause of heart failure. It typically occurs late in pregnancy or within the first 5 months following delivery^1^. PPCM is one of the leading causes of maternal morbidity and mortality worldwide, with an all-cause mortality rate of 9.8%, and accounts for 60% of the cases of cardiogenic shock in pregnancy and in the early postpartum period^2^. The global estimates of the incidence of PPCM vary geographically, ranging from 1 in 900 to 1 in 4000 deliveries in the United States, while Nigeria has reported the highest known incidence, accounting for 1 in 96 deliveries^3,4^. Despite its severity, the clinical features of PPCM mimic the normal physiological findings of pregnancy, thereby leading to diagnostic delays, increased risk of complications, and adverse clinical outcomes, including thromboembolism, maternal and neonatal mortality, and exacerbating LV dysfunction. Furthermore, data from the European Society of Cardiology reveals that LV function recovered in only 46% of the patients by 6 months, underscoring the need for a high index of clinical suspicion to facilitate early detection and prompt management of individuals with PPCM^5^. However, the current management strategies, centered on standard treatment of heart failure with reduced ejection fraction, are continuously evolving to more effectively address the unique aspects of PPCM. Personalized methods in PPCM therapy offer targeted and individualized approaches to diagnosis and treatment by leveraging genetic profiling, biomarker evaluations, and clinical phenotyping. Therefore, integrating genomic and phenotypic data establishes a novel clinical framework for personalized medicine in PPCM care, paving a new era of enhanced diagnostic precision and improved management strategies. This article provides insight into the potential role of precision medicine in managing PPCM.

## Main text

### Overview of pathophysiology and etiology of PPCM

PPCM is a type of systolic heart failure characterized by low ejection fraction, occurring in women during their final trimester of pregnancy or within the first 5–6 months postpartum^6^. This condition results in the weakening of the heart muscle, which can lead to cardiac failure if not treated^7^. The precise cause of PPCM is unknown, but it is believed to result from a combination of environmental and genetic factors, as well as pregnancy-related conditions such as pre-eclampsia^8^. Research suggests several other potential contributors to PPCM, including low selenium levels, viral infections, stress-activated cytokines, inflammation, autoimmune responses, abnormal reactions to hemodynamic stress, unbalanced oxidative stress, and the induction of antiangiogenic factors^9^. Risk factors for PPCM include first-time pregnancies (primiparity), hypertension, and multifetal pregnancies. While assisted reproduction techniques are not directly associated with PPCM, they may contribute indirectly through other risk factors^10^. Identifying predictive markers for PPCM is an ongoing research topic; however, some studies show that higher levels of natriuretic peptides, such as B-type natriuretic peptide (BNP) and N-terminal pro-B-type natriuretic peptide (NT-proBNP), may be effective in predicting the development of PPCM^7^.

In clinical practice, the symptoms of PPCM often mimic those of heart failure, including shortness of breath with exertion, orthopnea, paroxysmal nocturnal dyspnea, edema, and chest tightness. Common physical examination findings include tachypnea, tachycardia, increased jugular venous pressure, pulmonary rales, and peripheral edema^11^. Although rare, severe manifestations such as significant arrhythmias, cardiac arrest, thromboembolic events, and cardiogenic shock can also occur^3^. The primary challenge in diagnosing PPCM is distinguishing its symptoms from the regular physiological changes of pregnancy, such as fatigue, shortness of breath, and edema(12). One of the diagnostic criteria for PPCM is left ventricular systolic dysfunction with an ejection fraction of less than 45%^12^. Navigating these diagnostic obstacles emphasizes the significance of a thorough assessment and attentive monitoring, particularly in women with risk factors or concerning symptoms during the perinatal period.

### Current management strategies, limitations, and the need for personalized approaches in PPCM management

Conventional therapies for PPCM include a combination of drugs customized to the patient’s specific situation. Diuretics, such as thiazides, are safe and can be used for symptom alleviation; however, ACE inhibitors are not recommended during pregnancy due to their teratogenic potential^13^. Beta-blockers are used for conventional heart failure treatment and can be safely given throughout pregnancy and the postpartum period. Metoprolol is most usually administered because of its widespread usage in clinical settings and shorter half-life, allowing for a safe tolerance assessment. Vasodilators such as hydralazine should be used with caution for symptomatic relief^14^. Mineralocorticoid receptor antagonists, including spironolactone and eplerenone, are often prescribed for individuals with LVEF less than 40%^14^.

Guidelines for PPCM therapy emphasize the importance of optimizing preload and volume status through adequate diuresis and maintaining fluid balance intracellularly and extracellularly. Special considerations are necessary for prepartum PPCM therapy to avoid medications that can cross the placenta. For instance, diuretics like hydrochlorothiazide and furosemide are considered safe during pregnancy and breastfeeding but should be used with caution^7^. Given the heterogeneity and varied clinical manifestations of PPCM, personalized treatment approaches are crucial. A comprehensive evaluation, including a detailed medical history, physical examination, and diagnostic testing, is essential to tailor treatment strategies to each patient. A case study describes the effective use of bromocriptine in a patient with severe PPCM, highlighting the potential benefits of personalized therapy^15^.

The duration of pharmacotherapy is determined by the level of ventricular function recovery, with particular recommendations for each medicine based on pregnancy and lactation safety profiles and the patient’s LV function recovery state^14^. Overall, personalized methods in PPCM therapy constitute a paradigm shift toward tailored therapies that target patients’ unique requirements and features. By combining genetic insights, biomarker evaluations, and clinical phenotyping, personalized medicine can enhance risk classification, treatment effectiveness, and long-term results in PPCM.

### Genetic insights into PPCM

Genomic profiling in PPCM has recently gained attention due to its potential genetic predisposition. Multiple studies have shown that patients with PPCM complicated by cancer frequently have genetic variations associated with an increased risk of cancer^16^. Moreover, there is evidence suggesting that PPCM may occur in individuals with a positive family history of cardiomyopathies, indicating that genetic factors may play a role in unmasking a genetic form of the disease during pregnancy or the peripartum period^17^.

Genomic profiling has revealed a complex genetic landscape underlying PPCM, with notable similarities to other forms of cardiomyopathy, particularly dilated cardiomyopathy (DCM). Key genes implicated in PPCM include titin (TTN), Bcl2-associated athanogene 3 (BAG3), lamin A/C (LMNA), MYH6, MYH7, cardiac myosin binding protein C (MYBPC3), troponin C1 (TNNC1), troponin T2 (TNNT2), Duchene muscular dystrophy (DMD), and LAMP2^18–20^. Mutations in these genes affect various aspects of cardiac function: BAG3 influences protein folding, LMNA affects nuclear integrity, and several sarcomeric genes are crucial for muscle contraction. Truncating mutations in TTN, which codes for the large sarcomeric protein titin, are another common genetic factor. Moreover, X-linked genetic disorders linked to DMD and LAMP2 have also been associated with PPCM, underscoring the heterogeneous genetic basis of this condition^17^.

Furthermore, molecular pathways implicated in PPCM pathogenesis highlight disturbances in sarcomeric integrity and function, autophagy, cardiac conduction, electrophysiology, and oxidative stress response. Identifying these genetic variations through next-generation sequencing (NGS) technologies offers a gateway for personalized medicine approaches, including risk assessment, family screening, and tailored therapeutic strategies^17^. Despite the high prevalence of TTN truncating variants and diverse genetic etiology observed in PPCM, the mutation detection rate remains relatively low, suggesting the need for comprehensive genetic testing with larger gene panels to improve diagnostic accuracy and prognosis. Additionally, emerging evidence suggests potential roles for common genetic variants and heat shock protein (HSP) genes in PPCM pathogenesis, emphasizing the complex interplay of genetic factors in this disease^21^.

### Challenges and progress in biomarker discovery for PPCM

Diagnosing PPCM presents a significant challenge due to the absence of specific biomarkers. Several studies have looked into possible biomarkers for PPCM. Currently, NT-proBNP and brain-type natriuretic peptide (BNP) are being utilized clinically despite their specificity for PPCM being limited^22^. Cardiac troponin T (cTnT) and cardiac troponin I (cTnI) are indicators of heart muscle damage, but they lack extensive validation in PPCM. Soluble Fms-like tyrosine kinase-1 (sFlt-1) and placenta growth factor (PlGF) ratio also require further evaluation for diagnostic utility in patients with PPCM^23^. Additionally, 23-kDa prolactin (PRL) can be utilized for a better prognosis, while 16-kDa PRL, though indicative, lacks routine clinical applicability due to complex methodology^24^. Galectin-3 (Gal-3) is implicated in fibrosis and inflammation, but its diagnostic and prognostic value also requires extensive investigation. Procollagen type I and III N-terminal propeptides (PINP and PIIINP) signify collagen synthesis, potentially adding value to heart muscle remodeling in PPCM^25^. Overall, biomarker identification in PPCM remains an ongoing pursuit, with a need for validation studies to establish robust diagnostic and prognostic tools.

### Role of echocardiography and cardiovascular magnetic resonance in the improvement of PPCM diagnosis

Echocardiography stands as a critical imaging method for PPCM that can assess real-time cardiovascular dynamics during pregnancy^26^. It plays a significant role in distinguishing PPCM from other pregnancy-related cardiac conditions. Myocardial strain analysis, mainly through global longitudinal strain (GLS), is also a promising tool for early detection of myocardial impairment^27^. Furthermore, cardiovascular magnetic resonance (CMR) offers enhanced tissue characterization and thrombus identification, enhancing diagnostic precision^28^. Despite its potential benefits, CMR is not widely used in PPCM management, underscoring the need for greater integration into clinical practice to ensure precise diagnosis and monitoring.

### Pharmacogenomics in PPCM

Moreover, pharmacogenomics plays a significant role in tailoring drug therapies for PPCM, indicating that pharmacologically blocking prolactin could be a specific treatment for PPCM^29^. Additionally, combination therapy with beta-blockers, angiotensin-converting enzyme (ACE) inhibitors/angiotensin-receptor-blockers (ARBs), and bromocriptine has shown a high recovery rate in PPCM patients^30^. Furthermore, adding pentoxifylline to conventional therapy has also improved outcomes in patients with PPCM^31^. Bromocriptine therapy, along with standard heart failure treatment, has been associated with recovery from PPCM in some cases^32^.

### Accessible genetic testing for PPCM management

Accessibility and affordability of genetic testing are crucial in the management of PPCM. The advancements in next-generation sequencing (NGS) have made genetic analysis more accessible, allowing for the identification of genetic basis in patients with PPCM^33^. Incorporating sequencing approaches in people with cardiomyopathies to detect specific genetic variants into genetic testing can enhance test sensitivity. It can be helpful in early diagnosis and prompt treatment strategies. Despite the potential value of genetic testing in diagnosing genetic cardiomyopathies, the accessibility and cost-effectiveness of these tests remain essential for ensuring widespread utilization and improved outcomes.

Advancements in omics technologies, such as genomics and metabolomics, have revolutionized personalized medicine by providing comprehensive molecular insights into disease mechanisms. Developing predictive models and risk stratification tools using machine learning algorithms enables tailored treatment strategies based on individual patient profiles. Clinical trials play a crucial role in validating precision medicine approaches and guiding evidence-based decision-making in healthcare. Collaborative initiatives and global consortia facilitate knowledge sharing and resource pooling to advance precision medicine globally, ensuring the translation of research findings into clinical applications for the benefit of patients worldwide.

## Conclusion

In conclusion, PPCM poses a substantial risk for maternal morbidity and mortality, thus emphasizing the need for a comprehensive and personalized approach to its management. Owing to its varied clinical manifestations, the implications of precision medicine offer a promising avenue as it tailors treatment strategies based on individual patient profiles. Leveraging NGS technologies and biomarker identification enhances sensitivity along with improved diagnostic precision and therapeutic efficiency, ultimately leading to better patient outcomes. Further long-term studies and clinical trials are crucial to fully validate and gauge the effectiveness of precision medicine in PPCM management. Nevertheless, in the era of precision medicine, integrating these personalized therapeutic approaches into the clinical workflow can significantly revolutionize the landscape of PPCM management.

## Ethical approval

Ethics approval was not required for this review.

## Consent

Informed consent was not required for this review.

## Source of funding

The authors declare that they have no financial conflict of interest with regard to the content of this report.

## Author contribution

All authors have equally contributed to the manuscript and have approved the final manuscript to be published.

## Conflicts of interest disclosure

The authors declare that there no conflict of interest.

## Research registration unique identifying number (UIN)

Not applicable.

## Guarantor

Ajeet Singh.

## Data availability statement

Not applicable.

## Provenance and peer review

Not commissioned, externally peer-reviewed.
